# Estimate of inbreeding depression on growth and reproductive traits in a Large White pig population

**DOI:** 10.1093/g3journal/jkac118

**Published:** 2022-05-13

**Authors:** Yu Zhang, Yue Zhuo, Chao Ning, Lei Zhou, Jian-Feng Liu

**Affiliations:** National Engineering Laboratory for Animal Breeding; Key Laboratory of Animal Genetics, Breeding, and Reproduction, Ministry of Agriculture; College of Animal Science and Technology, China Agricultural University, Beijing 100193, China; National Engineering Laboratory for Animal Breeding; Key Laboratory of Animal Genetics, Breeding, and Reproduction, Ministry of Agriculture; College of Animal Science and Technology, China Agricultural University, Beijing 100193, China; Shandong Provincial Key Laboratory of Animal Biotechnology and Disease Control and Prevention, College of Animal Science and Technology, Shandong Agricultural University, Tai’an 271000, China; National Engineering Laboratory for Animal Breeding; Key Laboratory of Animal Genetics, Breeding, and Reproduction, Ministry of Agriculture; College of Animal Science and Technology, China Agricultural University, Beijing 100193, China; National Engineering Laboratory for Animal Breeding; Key Laboratory of Animal Genetics, Breeding, and Reproduction, Ministry of Agriculture; College of Animal Science and Technology, China Agricultural University, Beijing 100193, China

**Keywords:** Large White pigs, inbreeding coefficient, inbreeding depression, ROH, SNP

## Abstract

With the broad application of genomic information, SNP-based measures of estimating inbreeding have been widely used in animal breeding, especially based on runs of homozygosity. Inbreeding depression is better estimated by SNP-based inbreeding coefficients than pedigree-based inbreeding in general. However, there are few comprehensive comparisons of multiple methods in pigs so far, to some extent limiting their application. In this study, to explore an appropriate strategy for estimating inbreeding depression on both growth traits and reproductive traits in a Large White pig population, we compared multiple methods for the inbreeding coefficient estimation based on both pedigree and genomic information. This pig population for analyzing the influence of inbreeding was from a pig breeding farm in the Inner Mongolia of China. There were 26,204 pigs with records of age at 100 kg (AGE) and back-fat thickness at 100 kg (BF), and 6,656 sows with reproductive records of the total number of piglets at birth (TNB), and the number of alive piglets at birth (NBA), and litter weight at birth. Inbreeding depression affected growth and reproductive traits. The results indicated that pedigree-based and SNP-based inbreeding coefficients had significant effects on AGE, TNB, and NBA, except for BF. However, only SNP-based inbreeding coefficients revealed a strong association with inbreeding depression on litter weight at birth. Runs of homozygosity-based methods showed a slight advantage over other methods in the correlation analysis of inbreeding coefficients and estimation of inbreeding depression. Furthermore, our results demonstrated that the model-based approach (RZooRoH) could avoid miscalculations of inbreeding and inbreeding depression caused by inappropriate parameters, which had a good performance on both AGE and reproductive traits. These findings might improve the extensive application of runs of homozygosity analysis in pig breeding and breed conservation.

## Introduction

Inbreeding refers to the mating of individuals related to each other by ancestry, which increases the frequency of homozygotes in small populations (Falconer and Mackay 1996). Inbreeding is usually used to consolidate the hereditary traits of animals and establish breeds and lines in the domestication of several species. In addition, inbreeding increases the likelihood of being homozygous for recessive deleterious mutations, ultimately leading to inbreeding depression (ID) (Charlesworth and Willis 2009). ID on the fitness of individuals, production performance, and fertility has been frequently reported in livestock (Holyoake *et al.* 2006; Bijlsma and Loeschcke 2012; Markus *et al.* 2013; Ugnivenko 2018; Gutierrez-Reinoso *et al.* 2020).

The degree of inbreeding is measured by the coefficient of inbreeding (F), which is defined as the probability of the 2 alleles at any locus are identical by descent (IBD; Falconer and Mackay 1996). It is traditionally estimated from the pedigree (F_PED_). However, pedigree-based methods only give expected probabilities of IBD rather than their real value (Hill 1993) and are limited by inaccurate or missing pedigree information. With the extensive application of molecular genotyping technology, genomic data are widely used to estimate the inbreeding coefficient (Leutenegger *et al.* 2003), such as F based on the percentage of homozygous(F_PH_), derived from the genomic relationship matrix (F_GRM_; Yang *et al.* 2010) and the observed vs expected number of homozygous genotypes (F_HOM_). Compared with F_PED_, the above SNP-based measures could avoid the effect of genealogical error and detect ID more effectively in simulation studies (Wang 2016). Besides, long runs of homozygous genotypes, known as runs of homozygosity (ROHs), represent autozygosity (McQuillan *et al.* 2008). The inbreeding coefficient based on ROH (F_ROH_) was reported that it was more accurate for assessing individual inbreeding levels than other inbreeding coefficient estimators in recent studies (Schäler *et al.* 2020; Shi *et al.* 2020). The criteria of this rule-based method had to be optimized for different populations and the genotyping technology, but the software RZooRoH could detect ROHs easily without a predefined threshold (Bertrand *et al.* 2019).

ID was usually associated with the reduced expression of dominance effects, which was also used in genomic models to estimate dominance and epistatic genetic variance (Varona *et al.* 2018; Vitezica *et al.* 2018). This inclusion of ID could improve the predictive ability for the total number of piglets at birth (TNB) in a pig’s cross-breeding program (Xiang *et al.* 2016). Accurate estimates of inbreeding and ID could also improve the efficiency of the mate allocation strategies and breeding programs (Zhang *et al.* 2015; González-Diéguez *et al.* 2019).

In pigs, many studies suggested that an increase of the inbreeding coefficient resulted in a significant reduction of reproductive traits, including the TNB, the number of alive piglets at birth (NBA), and gestation length (Farkas *et al.* 2007). In addition, the increased inbreeding negatively affected growth and production performance, such as lean meat percentage, average daily gain, and growth traits at pre- or weaning (Vigh *et al.* 2008; Gowrimanokari *et al.* 2019). More recent studies provided significant evidence that TNB was associated with ID in a Korean native pig population (Kim *et al.* 2019) and an Iberian pig population (Casellas *et al.* 2019). According to a meta-analysis of different studies, SNP-based estimates of ID were more powerful than pedigree-based estimates (Doekes *et al.* 2021). Silió *et al.* (2013) reported that the daily growth rate decreased by 0.007 kg/day and weight at 90 days decreased by 0.308 kg with a 10% increase of genomic inbreeding coefficients in Iberian pigs. For TNB and NBA, a 10% increase in F_HOM_ on chromosome 13 caused a decline of 0.121 and 0.117, respectively (Saura *et al.* 2015). However, the above studies on pigs were incomplete for lack of the application of new methods, such as F_ROH_ based on the underlying hidden Markov model. On the other hand, they were limited to small groups and lacked comprehensive comparison with multiple methods. This study aimed to compare several methods for estimating the inbreeding coefficient based on pedigree and SNP information and investigate a suitable method for estimating ID on different traits in a Large White pig population.

## Materials and methods

### Animals and phenotypes

The data were collected from a pig breeding farm in Inner Mongolia, China. The dataset contained a pedigree with 52,789 Large White pigs, of which 27,341 animals had at least 1 trait. There were 26,204 animals with growth traits and 6,656 animals with reproductive traits (21,680 records), of which 5,519 sows have both growth and reproductive phenotypes. All animals were born between 2013 and 2020.

The evaluated growth traits were age at 100 kg (AGE) and back-fat thickness at 100 kg (BF), and they were adjusted according to the National Swine Performance Recording Standards of China (Supplementary File 1). Reproductive traits included TNB, NBA, and litter weight at birth.

### Genomic data

Genotyping was conducted using an SNP chip named CAU50K, including 43,832 SNPs (The map version used for SNP positions is Sscrofa10.2). Quality control of the genotype data was implemented by PLINK (Purcell *et al.* 2007) using the criteria [marker genotype deletion rate <1%, sample genotype deletion rate <1%, minor allele frequency (MAF) >0.01, and Hardy-Weinberg Equilibrium (HWE) > 10e−4). Finally, a total of 1,600 pigs and 35,304 SNPs on autosomes remained for subsequent analysis. There were only 1,599 pigs with both genotype and pedigree information, of which 1,592 animals had growth phenotypes, and 1,187 sows had reproductive phenotypes (5,566 records).

### Estimation of inbreeding coefficients

The pedigree-based inbreeding coefficients with an average depth of 8.23 generations were estimated by the R package “Pedigree” (Coster 2012). F_PED(total)_ was estimated from 27,341 animals with at least 1 trait. Due to missing pedigree information, F_PED_ was only estimated for the 1,599 animals with both genotype and pedigree information.

Genomic inbreeding coefficients were estimated by 4 different estimators, including the percentage of homozygous SNPs (F_PH_), the genomic relationship matrix (F_GRM_), and the observed vs expected number of homozygous genotypes (F_HOM_), and runs of homozygosity (F_ROH_). Their detailed computation strategies were as follows:

1. F_PH_ refers to the proportion of homozygous genotypes of all genotypes.
2. Genomic inbreeding coefficients derived from the genomic relationship matrix (F_GRM_) were calculated by GCTA soft (Yang *et al.* 2011). To explore whether pruning data for stringent MAF may decrease F_GRM_, F_GRM2_ were estimated from 32,840 SNPs when MAF was set at 0.05 as a comparison.
3. F_HOM_, which is based on the observed vs expected number of homozygous genotypes, was estimated using PLINK soft (Purcell *et al.* 2007). The formula is $F_{\text{HOM}}=\frac{O-E}{L-E}$, where O is the number of observed homozygotes, E is the number expected by chance, and L is the number of genotyped autosomal SNPs. According to the PLINK software recommendation, F_HOM(pruned)_ was also estimated after SNP pruning (with a sliding window of 50 SNPs, shifting the window 5 SNPs forward and removing SNPs with *r*^2^ > 0.5; –indep-pairwise 50 5 0.5) based on linkage disequilibrium (LD), with 11,270 SNPs remaining.
4. F_ROH_ was based on ROH, which was detected based on the different criteria using PLINK v1.9 and on a hidden Markov model framework using RZooRoH (Bertrand *et al.* 2019). Most studies estimated the inbreeding coefficient based on the length of ROH (McQuillan *et al.* 2008), which was used for ROH_1, ROH_2, and ROH_3 in this study. Besides, the inbreeding coefficient was also determined as the sum of SNPs in the ROHs divided by the total number of SNPs, and it was marked F_ROH(N)_. A minimum density of 1 SNP per 80 kb was set to prevent low SNP density from affecting ROH length. The formula (Lencz *et al.* 2007; Purfield *et al.* 2012) determined the minimal number of SNPs in an ROH. As a result, both the minimal number of SNPs per ROH and per window were set to 66 (the corresponding PLINK parameters for ROH_1 are “-homozyg-density 80,” “–homozyg-snp 66,” and “-homozyg-window-snp 66”). To correct for false-positive ROH caused by LD, ROH_2 included the stringent criteria for setting the minimum ROH length to 500 kb on the base of ROH_1. Moreover, the maximal genome coverage is the proportion of the maximal detectable ROH length over the length of the genome and is used to reflect the ROH analysis’ validity. To compare the effectiveness of this indicator, we set the minimum density of 1 SNP was set to 70 kb in ROH_3.

The model-based ROH detection method used RZooRoH. The software implements an efficient and accurate approach based on the hidden Markov model to identify homozygous-by-descent (HBD) segments associated with ROHs. We set a 2-state model (1R model), which estimated the probability of 2 consecutive markers to be either HBD or non-HBD. The genotyping error rate was set to 0.025%, as suggested by Ferencakovic *et al.* (2013). Finally, F_ROH(model)_ was calculated as the proportion of the genome in HBD classes.

The inbreeding coefficients estimated by these methods were compared using Pearson’s correlation with the data set of 1,599 pigs with both genotype and pedigree information.

### Estimation of ID

The linear mixed model estimated ID on growth and reproductive traits. ID was estimated separately for AGE and BF using the following bivariate animal model:

$$\left[\begin{array}{c} y_{1}\\ y_{2} \end{array}\right]=\left[\begin{array}{cc} X_{1} & 0\\ 0 & X_{2} \end{array}\right]\left[\begin{array}{c} \beta_{1}\\ \beta_{2} \end{array}\right]+\left[\begin{array}{cc} f_{1} & 0\\ 0 & f_{2} \end{array}\right]\left[\begin{array}{c} b_{1}\\ b_{2} \end{array}\right]+\left[\begin{array}{cc} Z_{1} & 0\\ 0 & Z_{1} \end{array}\right]\left[\begin{array}{c} \alpha_{1}\\ \alpha_{2} \end{array}\right]+\left[\begin{array}{cc} W_{1} & 0\\ 0 & W_{1} \end{array}\right]\left[\begin{array}{c} c_{1}\\ c_{2} \end{array}\right]+\left[\begin{array}{c} e_{1}\\ e_{2} \end{array}\right]$$

where the column vectors of observations $y_{1}\;$and $y_{2}$ represent data of AGE and BF, $\beta_{1}$ and $\beta_{2}$ are vectors of fixed effects on 2 traits, including the gender, the strain, the year-season at birth, and the number of alive littermates. Besides the above common, $\beta_{1}$ for AGE also includes birth weight as a covariate, and $\beta_{2}$ for BF includes birth parity. $b_{1}\;$and $b_{2}$ are the regression coefficients on $f_{1}$ and $f_{2}$, and they are vectors of inbreeding coefficients for AGE and BF, respectively. $\alpha_{1}$ and $\alpha_{2}$ are vectors of additive genetic effects for each animal. $c_{1}$ and $c_{2}$ are vectors of common litter environment effects, and $e_{1}$ and${\;e}_{2\;}$are vectors of random residuals. $X_{i}$, $Z_{i}$, and $W_{i}$ are the incidence matrices relating records of the ith trait to fixed effects ($\beta_{i}$), additive genetic effects ($\alpha_{i}$), and common litter environment effects ($c_{i}$), respectively. Moreover, the (co)variance structure of random effects of this model is as follows:

$$\text{V}\left[\begin{array}{c} \begin{array}{c} \mu_{1}\\ \mu_{2}\\ c_{1} \end{array}\\ \begin{array}{c} c_{2}\\ e_{1}\\ e_{2} \end{array} \end{array}\right]=\left[\begin{array}{ccc} \begin{array}{cc} A\sigma_{\alpha_{1}}^{2} & A\sigma_{\alpha_{12}}\\ A\sigma_{\alpha_{12}} & A\sigma_{\alpha_{2}}^{2} \end{array} & \begin{array}{cc} 0 & 0\\ 0 & 0 \end{array} & \begin{array}{cc} 0 & 0\\ 0 & 0 \end{array}\\ \begin{array}{cc} 0 & 0\\ 0 & 0 \end{array} & \begin{array}{cc} I\sigma_{c_{1}}^{2} & I\sigma_{c_{12}}\\ I\sigma_{c_{12}} & I\sigma_{c_{2}}^{2} \end{array} & \begin{array}{cc} 0 & 0\\ 0 & 0 \end{array}\\ \begin{array}{cc} 0 & 0\\ 0 & 0 \end{array} & \begin{array}{cc} 0 & 0\\ 0 & 0 \end{array} & \begin{array}{cc} I\sigma_{e_{1}}^{2} & I\sigma_{e_{12}}\\ I\sigma_{e_{12}} & I\sigma_{e_{2}}^{2} \end{array} \end{array}\right]$$

where A is the relationship matrix; $\sigma_{\alpha_{i}}^{2}$ is additive genetic variances for the ith trait, $\sigma_{c_{i}}^{2}$ and $\sigma_{e_{i}}^{2}$ are variances of common litter environment and residual effects for the ith trait, respectively; $\sigma_{\alpha_{12}}$, $\;\sigma_{c_{12}}$ and $\;\sigma_{e_{12}}$ are covariances of additive genetic effects, common litter environment, and residual effects between 2 traits.

In practice, the reproductive traits usually use the repeatability model to estimate breeding value, so we took the different strategies from growth traits to estimate ID on TNB, NBA, and litter weight at birth, as follows:

y=Xβ+fb+Zα+Wp+e

where **y** is a vector of observations, β is a vector of fixed effects, including the year-season at birth and parity. b is the regression coefficient on f, which is a vector of inbreeding coefficients. X is the incidence matrix relating records to fixed effects (β). α is a vector of additive random animal effects distributed with means of zero and variance ${\mathrm{A\sigma}}_{\alpha }^{2}$; **A** is the relationship matrix. **Z** is the incidence matrix relating records to additive random animal effects; p is a vector of random permanent environmental effects, to which incidence matrix W relates records; e is a vector of random residual effects. It is assumed that the permanent environmental effects and residual effects are independently distributed with means of zero and variance $I\sigma_{p}^{2}\;$and ${I\sigma }_{e}^{2}$, respectively.

The statistical analysis was performed with the DMU program package (Madsen and Jensen 2008), and a *t*-test was used to obtain *P*-values.

## Results

### ROH and inbreeding coefficient

**Fig. 1.**
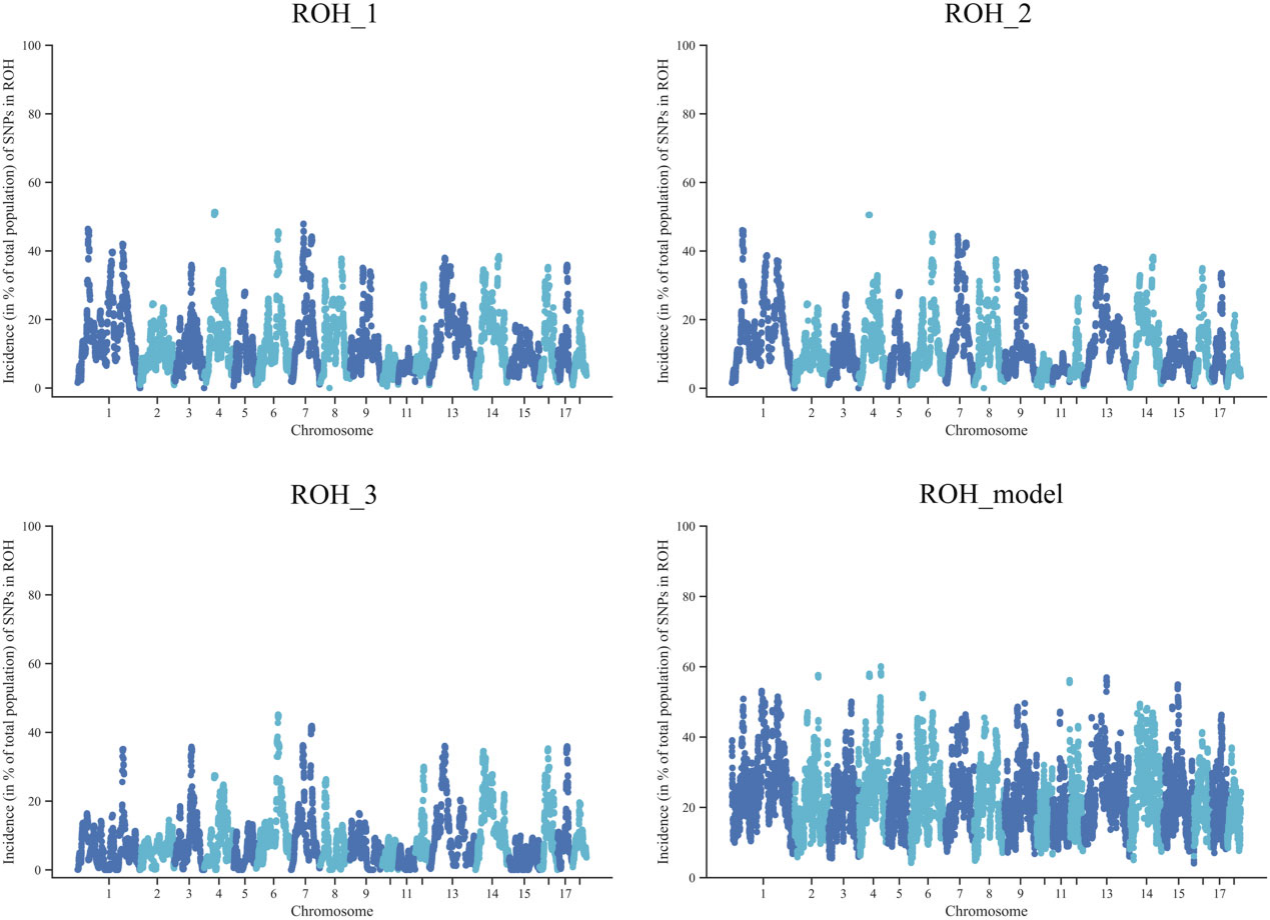
Incidence plots of SNPs in an ROH on chromosomes. The ROH_1 was based on Meyermans’s recommendation. More strict parameters about the length of an ROH (>5 Mb) for ROH_2 and a minimum density of 1 SNP per ROH (<70/kb) for ROH_3 were set. ROH_model used the model-based software RZooRoH.

We used 3 kinds of parameter settings in PLINK and a model-based method in RZooRoH to detect ROHs in a Large White pig population with 1,600 pigs genotyped. The numbers of ROHs were 47,133, 39,529, and 31,721, with an average length of 10,827.20, 12,049.43, and 9,228.63 kb for the 3 rule-based methods for ROH_1, ROH_2, and ROH_3, respectively. RZooRoH detected 208,211 ROH segments with an average of 4,194.77 kb, which was over 4 times the number detected by PLINK. Using PLINK, the length of most ROHs was between 5 and 10 Mb, whereas 78% of homozygous segments were short (less than 5 Mb) using RZooRoH (Supplementary Fig. 1). The distribution of ROH incidence per SNP for ROH_1 was similar to ROH_2 (Fig. 1). However, some ROH islands (highly homozygous regions) were ignored for ROH_3, especially on SSC1, SSC4, and SSC9. Also, Fig. 1 clearly showed that the model-based approach could detect more SNPs in ROHs than the rule-based method.

**Fig. 2.**
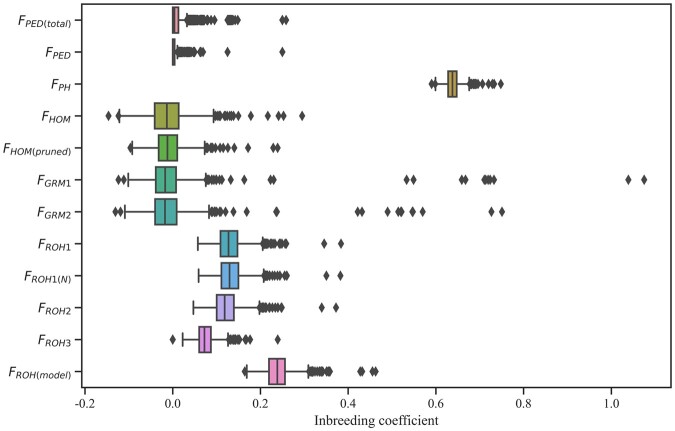
Box plots of inbreeding coefficients estimated with different methods for data sets. F_PED(total)_ was estimated from the total animals of 27,341, and F_PED_ included 1,599 animals with both genotype and pedigree information. F_PH_ = the proportion of homozygous genotypes of all genotypes; F_GRM_ = inbreeding from genomic relationship matrix, F_GRM1_ and F_GRM2_ were from pruning data for MAF (0.01) and MAF (0.05); F_HOM_ = inbreeding coefficients based on the observed vs expected number of homozygous genotypes, F_HOM(pruned)_ was from SNP pruned data based on LD; Under the different settings (ROH_1, ROH_2, and ROH_3), F_ROH_ was estimated based on runs of homozygosity; F_ROH(N)_ was determined as the sum of SNPs in the ROHs divided by the total number of SNPs; F_ROH(model)_ was estimated using the model-based software RZooRoH.

F_PED(total)_ was estimated from all animals with phenotype and pedigree information, and F_PED_ was estimated from the animals with genotype and pedigree information. The average F_PED(total)_ was equal to the average F_PED_ at 0.01 (see Supplementary Table 1), and the average genomic inbreeding coefficients using different methods varied widely. The average F_PH_ was 0.64 ± 0.02 ranging from 0.56 to 0.73. While the range of F_HOM(pruned)_ was smaller after pruning based on LD (Fig. 2), both the average F_GRM_ and F_HOM_ were −0.01 no matter whether the data were pruned or not. The average inbreeding coefficients with the rule-based approach were about 0.13, and the standard deviations were 0.03, except for F_ROH3_. F_ROH3_, with an average of 0.07 ± 0.02, was lower than the other 2 estimates. The same trends were also observed in detecting ROHs. The average F_ROH(model)_ was 0.24 ± 0.03 ranging from 0.16 to 0.46, which was higher than other estimates based on ROHs.

**Fig. 3.**
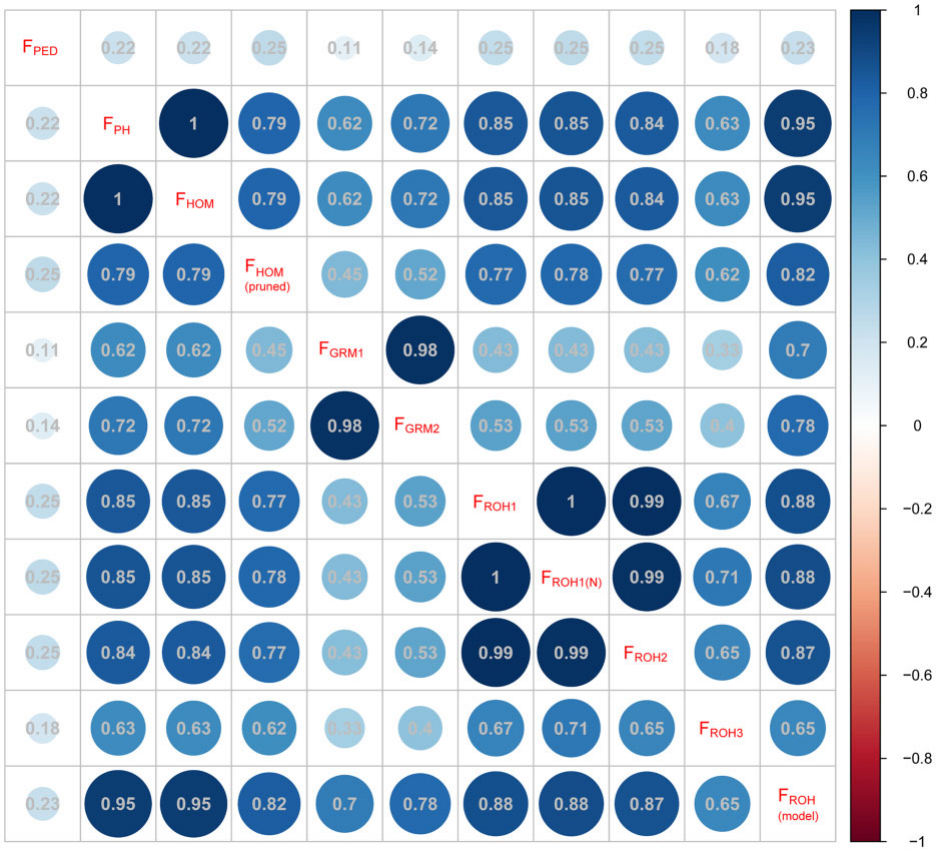
Correlations between different estimates of inbreeding. Every pair of inbreeding coefficients was related significantly (*P* < 0.01, Pearson correlation). F_PED_ only included 1,599 animals with both genotype and pedigree information. F_PH_ = the proportion of homozygous genotypes of all genotypes; F_GRM_ = inbreeding from genomic relationship matrix, F_GRM1_ and F_GRM2_ were from pruning data for MAF (0.01) and MAF (0.05); F_HOM_ = inbreeding coefficients based on the observed vs expected number of homozygous genotypes, F_HOM(pruned)_ was from SNP pruned data based on LD; under the different settings (ROH_1, ROH_2, and ROH_3), F_ROH_ was estimated based on ROH; F_ROH(N)_ was determined as the sum of SNPs in the ROHs divided by the total number of SNPs; F_ROH(model)_ was estimated using the model-based software RZooRoH.

Every pair of inbreeding coefficients was considered statistically significant at the 0.01 level. Correlations between the pedigree-based and genomic inbreeding coefficients were weak, ranging from 0.11 to 0.25 (Fig. 3). The highest correlations with F_PED_ were F_HOM(pruned)_, F_ROH1,_ and F_ROH2_ (*r* = 0.25), followed by F_ROH(model)_ (*r* = 0.23). The lowest correlation with F_PED_ was F_GRM1_ calculated from the loose criterion for MAF (0.01). Except for F_GRM_, there were strong correlations between other genomic F. Although the correlation coefficient between F_HOM_ and F_HOM(pruned)_ was relatively lower (just 0.79), the correlations using the same approach and different parameters were strong. Compared with F_HOM(pruned)_, F_HOM_ had a lower association with F_PED_ and a higher association with genomic inbreeding estimates. In contrast, the correlation coefficient between F_GRM_ from pruning data for MAF > 0.01 and MAF > 0.05 was 0.98. Compared to F_GRM1_ (MAF > 0.01), F_GRM2_ (MAF > 0.05) had relatively strong correlations with other genomic inbreeding coefficients. For ROH-based methods, the correlation coefficients among F_ROH1_, F_ROH1(N)_, and F_ROH2_ were around 1. Also, F_ROH(model)_ using RZooRoH had the highest correlation with other genomic F, followed by F_ROH1_/F_ROH2_ and F_ROH3_.

### Inbreeding depression

**Fig. 4.**
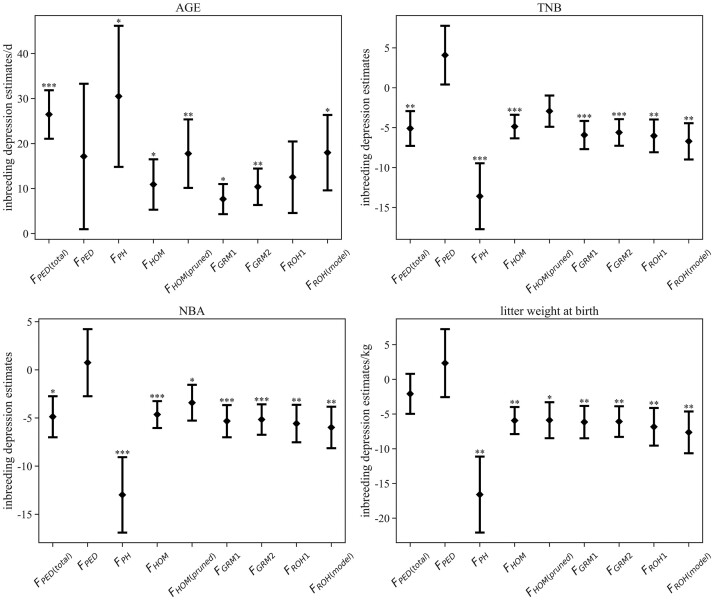
Estimates of ID on growth and reproductive traits using different methods (error bar refers to the standard error). *P-value < 0.05, **P-value < 0.01, ***P-value < 0.001 ID with F_PED(total)_ were estimated from the total animals with pedigree information, and F_PED_ only included animals with both genotype and pedigree information. F_PH_ = the proportion of homozygous genotypes of all genotypes; F_GRM_ = inbreeding from genomic relationship matrix, F_GRM1_ and F_GRM2_ were from pruning data for MAF (0.01) and MAF (0.05); F_HOM_ = inbreeding coefficients based on the observed vs expected number of homozygous genotypes, F_HOM(pruned)_ was from SNP pruned data based on LD; For ROH_1, F_ROH_ was estimated based on runs of homozygosity; F_ROH(model)_ was estimated using the model-based software RZooRoH.

All the results of descriptive statistical analysis of traits and estimates of the ID are shown in the Supplementary materials (Supplementary Tables 2 and 3). When F_PED_ was used as a covariate in the model, no statistically significant results were obtained. However, F_PED(total)_ had a statistically significant effect on AGE, with each 10% increase of F_PED(total)_ extending 2.6 ± 0.5 days. Furthermore, an increase of 10% in F_PH_, F_HOM_, and F_GRM1_ was associated significantly with the extension of AGE by 3.0 ± 1.6, 1.1 ± 0.6, and 0.8 ± 0.3 days, respectively. For F_HOM_, LD-based SNP pruning declined the *P*-value and increased the estimate of ID on AGE, and there was a similar trend using the strict criteria (MAF < 0.05, F_GRM2_). An increase of 10% in F_ROH(model)_ caused the extension of AGE by 1.8 ± 0.8 days, while not all inbreeding coefficients using ROH-based measures had a significant ID effect, such as F_ROH1_. From the error bar plot (Fig. 4), it was evident that the maximum estimates of ID on AGE for F_HOM_, F_GRM1_, and F_GRM2_ were lower than the minimum estimate in F_PED(total)_. The estimates of ID on AGE with F_PH_, F_ROH(model)_, and F_HOM(pruned)_ were close to the result for F_PED(total)_. Nevertheless, compared to the other 2 methods, the disadvantage with F_PH_ was a relatively large standard error of estimates. All pedigree-based and genomic inbreeding coefficients were hardly associated significantly with back-fat thickness. As a result, the error bar plot of estimates for BF was not shown in Fig. 4.

F_PED(total)_ significantly affected TNB and NBA, which is different from F_PED_. For litter weight at birth, there was no ID effect for both F_PED_ and F_PED(total)_. An increase of 10% in F_PED(total)_ contributed to a decrease of 0.5 ± 0.2 piglets both for TNB and NBA. Most genomic inbreeding coefficients significantly affected 3 reproductive traits. Specifically, the corresponding estimates of ID on TNB, NBA, and litter weight at birth were 1.4 ± 0.4 piglets, 1.3 ± 0.4 piglets, and 1.7 ± 0.5 kg, with a 10% increase in F_PH_. Moreover, the maximum estimate with F_PH_ was much lower than the minimum estimate with F_PED(total)_ (Fig. 4). The estimates of ID with F_HOM_ and F_GRM1_ were similar, with approximately 0.5 piglets, 0.5 piglets, and 0.6 kg, with a 10% increase. The result differing from ID on AGE was that SNP pruning resulted in a higher *P*-value of ID on 3 reproductive traits and a lower absolute value. Besides, the estimates with F_GRM1_ and F_GRM2_ were very close, of which the difference is less than 0.32. Both F_ROH1_ and F_ROH(model)_ had significant effects on reproductive traits, and corresponding estimates were about 0.6 piglets (TNB), 0.6 piglets (NBA), and 0.7 kg (litter weight at birth), respectively.

## Discussion

### ROH and inbreeding coefficient

When the minimal number of SNPs per ROH was set to 66, calculated by Lencz’s formula, short ROHs (<5 Mb) had little effect on the detection of genome regions with ROH hotspots overlapping and estimates of inbreeding (*r* = 0.99). However, the long homozygous regions in the genome might be ignored with strict criteria of minimal density (ROH_3). Combined with the distribution of ROHs on chromosomes (Supplementary Fig. 2), it was evident that 2 ROH hotspots for ROH_2 were absent for ROH_3, located on SSC1 (the position from 48.5 to 54.8 Mb) and SSC4 (the position from 48.2 to 53.4 Mb), respectively. The same gene region on SSC4 was also detected in pig populations of Poland (Szmatoła *et al.* 2020). Furthermore, gene enrichment analysis indicated that the genes on 2 ROH hotspots are primarily associated with metabolic process and lyase activity. Moreover, some genes, such as *NT5E*, *COL9A1*, *LMBRD1*, *CA3*, *ATP6V0D2*, and *SLC7A13*, were associated with the absorption and metabolism of nutrients.

The average pedigree-based inbreeding coefficient with an average depth of 8.2 generations was small, and there were weak correlations between F_PED_ and other genomic F in agreement with the study of Shi *et al.* (2020). In addition, the average F_PH_ was near to the study for Large White pigs (Zanella *et al.* 2016), and the averages of F_GRM_ and F_HOM_ were negative. The correlation between F_PED_ and F_GRM_ was only 0.11, which is consistent with local pigs of northern Germany (Schäler *et al.* 2020) and Simmental cattle (Marras *et al.* 2015). It was reported that correlations between F_GRM_ and other genomic F were lower than that among other genomic F (Mastrangelo *et al.* 2016; Schäler *et al.* 2020). Our study also confirmed this point. Because there was no definite criterion to judge the most suitable method representing the actual inbreeding level of a population, the method having a relatively high association with both pedigree-based and most genomic methods was considered a good estimator. It should be noted that the highest correlations with F_PED_ were F_HOM_ after SNP pruning based on LD (F_HOM(pruned)_) and F_ROH_. When SNP pruning had a significant impact on estimating inbreeding, the disadvantage of F_HOM(pruned)_ was a lack of guidelines on the applicability of SNP-pruning parameters. However, according to the practical guide to ROH analysis offered by Meyermans *et al.* (2020), it is easy to choose a proper parameter to estimate F_ROH_. Moreover, the correlation coefficients of most ROH-based methods with other genomic methods were high, especially F_ROH(model)_. These results suggested that F_ROH_ had a distinct advantage in estimating the level of inbreeding under appropriate parameter settings or using the model.

The F_ROH_ was obtained by 2 computing methods, based on the number of SNPs in ROH and the length of ROHs, both used in previous literature (Pryce *et al.* 2014; Sams and Boyko 2019). The high correlation between F_ROH1_ and F_ROH(N)_ (*r* = 1) indicated that the 2 methods tended to be the same. Although the minimal density was recommended to 70 kb/SNP (ROH_3) by Meyermans *et al.* (2020), it also mentioned that the maximal genome coverage was a necessary validator in ROH analysis. In this study, this validator for ROH_3 was just 61.37%. When a looser standard with 80 kb/SNP (ROH_1 and ROH_2) was used, the maximal genome coverage was 99.7%. The low maximal genome coverage also caused the poor performance. Besides a low correlation with F_ROH1_ (*r* = 0.67), F_ROH3_ correlated poorly with F using other measures. It suggested that genome coverage was essential to estimating the level of inbreeding. The inbreeding coefficient of the model-based approach (RZooRoH) correlated with the estimates of the rule-based approach (*r* = 0.65–0.88), but its correlations with F_PH_ and F_HOM_ were higher. This result was probably because the rule-based approach ignored some short ROHs less than 5 Mb in length, which could be misidentified as identity by state.

### Inbreeding depression

We could observe the statistically significant ID associated with pedigree-based inbreeding coefficients from 27,341 animals, but not when intersection with phenotypes and genotypes was calculated. For AGE, TNB, and NBA, a 10% increase in F_PED(total)_ led to adverse effects of 2.6 ± 0.5 days, 0.5 ± 0.2 piglets, and 0.5 ± 0.2 piglets. These estimates for TNB and NBA were higher than that in Iberian pigs (Saura *et al.* 2015). In addition, F_PED(total)_ had no effect on BF and litter weight at birth. Although a small dataset was not conducive to estimating ID, genomic measures had a good performance without an amount of information.

Compared with F_PED(total)_, using F_PH_ generated a relatively large standard error and might overestimate ID on both growth and reproduction traits. For AGE, the estimates of ID, with F_GRM_ for strict MAF criterion (MAF < 0.05) and F_HOM_ after SNP pruning based on LD, had more power. Because the loose criterion of MAF and no SNP pruning contributed to overestimating the inbreeding of certain animals, ID was finally underestimated. If F derived from the genomic relationship matrix is used to estimate ID on growth traits, the MAF should be set to 0.05 instead of 0.01. In addition, the estimate of ID on AGE with F_ROH(model)_ was higher than with F_GRM2_ and near to F_HOM(pruned)_. However, F_ROH1_ had no significant effect on AGE. While there is no obvious evidence that model-based F_ROH_ is the best method of ID on AGE among SNP-based methods, the difference between F_ROH(model)_ and F_PED(total)_ is comparatively small.

The estimates of ID on reproductive traits among various genomic methods were much closer than estimates on growth traits, except for F_HOM(pruned)_ and F_PH_. The results of ID using F_GRM_ and all ROH-based F estimating for TNB and NBA were similar to studies in Iberian pigs (Saura *et al.* 2015; Silió *et al.* 2016) and lines from Genus PIC (Varona *et al.* 2018). Using F_PH_ was more likely to be overestimated ID on reproductive traits, while using F_HOM(pruned)_ may underestimate it. Only SNP-based F revealed a strong association with ID on litter weight at birth, ranging from 0.5 to 0.8 kg per 10% increase. In contrast with AGE, ID on reproductive traits with SNP-based F had a lower *P*-value than pedigree-based F. There were many reasons for these differences, such as errors in pedigree records, expected or actual IBD, and the number of samples. Anyhow, both our study and studies in dairy cattle (Pryce *et al.* 2014; Martikainen *et al.* 2017) found that the use of pedigree information might underestimate ID on female fertility.

In summary, we found pedigree-based and SNP-based measures had significant effects on AGE, TNB, and NBA, except for BF. Especially in terms of litter weight at birth, only SNP-based F revealed a strong association with ID. The estimate of ID in F_PED_ was susceptible to the number of samples, but the genomic method had a better performance with a smaller data set. Most genomic methods tended to provide a more similar estimation of ID on reproductive traits than growth traits. From the practical point of view, the method based on the hidden Markov model (RZooRoH) did not need to worry about the impact of parameters and performed well on both AGE and reproductive traits in the estimate of inbreeding and ID. These findings might improve the extensive application of ROH analysis in pig breeding and breed conservation.

## Data availability

Supplementary File 1 contains a list of all supplemental files. Supplementary File 2 contains supplemental files, figures, and tables. Supplementary Files 3 and 4 contain phenotype information of growth traits and reproductive traits for Large White pigs, respectively. In addition, Supplementary File 5 contains the pedigree of Large White pigs in this study. Supplementary Files 6 and 7 contain genotype and maker location information in PLINK .ped/.map formation for Large White pigs. Supplementary material is available at figshare (https://doi.org/10.25387/g3.17207063).

## Funding

This study was funded by the National Natural Science Foundation of China (NSFC) under grants Nos. 31661143013, 31790414 and 31972563, and China Agriculture Research System (No. CARS-35-01A).

## Conflicts of interest

No potential conflict of interest was reported by the authors.
